# Glycine-Serine-Threonine Metabolic Axis Delays Intervertebral Disc Degeneration through Antioxidant Effects: An Imaging and Metabonomics Study

**DOI:** 10.1155/2021/5579736

**Published:** 2021-08-25

**Authors:** Xiaolin Wu, Chang Liu, Shuai Yang, Nana Shen, Yan Wang, Youfu Zhu, Zhaoyang Guo, Shang-you Yang, Dongming Xing, Houxi Li, Zhu Guo, Bohua Chen, Hongfei Xiang

**Affiliations:** ^1^Department of Orthopedics, The Affiliated Hospital of Qingdao University, Qingdao, China 266003; ^2^Department of Rehabilitation, The Affiliated Hospital of Qingdao University, Qingdao, China 266000; ^3^School of Medicine-Wichita, University of Kansas, 929 N St. Francis Street, Wichita, KS, USA 67214; ^4^Cancer Institute, The Qingdao University, Qingdao, China 266003; ^5^School of Life Sciences, Tsinghua University, Beijing, China 100084; ^6^Shandong First Medical University, Taian 271016, China

## Abstract

Although intervertebral disc degeneration (IDD) can be described as different stages of change through biological methods, this long and complex process cannot be defined in stages by single or simple combination of biological techniques. Under the background of the development of nuclear magnetic resonance (NMR) technology and the emerging metabonomics, we based on animal models and expanded to the study of clinical human degeneration models. The characteristics of different stages of IDD were analyzed by omics. Omics imaging combined with histology, cytology, and proteomics was used for screening of the intervertebral disc (IVD) of research subjects. Furthermore, mass spectrometry nontargeted metabolomics was used to explore profile of metabolites at different stages of the IDD process, to determine differential metabolic pathways and metabolites. NMR spectroscopy was used to qualitatively and quantitatively identify markers of degeneration. NMR was combined with mass spectrometry metabolomics to explore metabolic pathways. Metabolic pathways were determined through protein molecular biology and histocytology of the different groups. Distinguishing advantages of magnetic resonance spectroscopy (MRS) for analysis of metabolites and effective reflection of structural integrity and water molecule metabolism through diffusion tensor imaging (DTI) were further used to verify the macrometabolism profile during degeneration. A corresponding model of *in vitro* metabolomics and *in vivo* omics imaging was established. The findings of this study show that a series of metabolic pathways associated with the glycine-serine-threonine (Gly-Ser-Thr) metabolic axis affects carbohydrate patterns and energy utilization efficiency and ultimately delays disc degeneration through antioxidant effects.

## 1. Introduction

Lumbar disc degeneration causes lumbar disc herniation, lumbar spinal stenosis, and other lumbar disc degeneration diseases [1]. Degenerative disc disease involves genetic, mechanical, immune, metabolic, and other complex physiological processes [2–4].

The intervertebral disc (IVD) is located between the vertebral bodies. It facilitates in intervertebral motion and distributes compressive load in adjacent vertebral bodies [5]. IVD consists of nucleus pulposus (NP), annulus fibrosus (AF), and endplates (EP) forming a relatively closed organ [6]. It is the largest hypovascular unit in the human body [7] and an immune-exempt organ [8]; therefore, its special anatomical structure determines the pathophysiological specificity of IVD tissue [9]. The process and mode of degeneration of IVD are different from other tissues and organs. Loss of NP cells through degeneration and necrosis is the main feature of intervertebral disc degeneration (IDD). The extracellular matrix (ECM) component [10] and content of the type II collagen-based ECM secreted show significant changes during degeneration. ECM is the morphological maintenance structure. The live environment of NP cells and its metabolic regulation dominate the degeneration process.

Clinical diagnosis of IDD is mainly based on magnetic resonance imaging (MRI) as the first noninvasive examination. Degree of degeneration is defined by Pfirrmann grading, which is based on T_2_WI images [11]. The overall shape and signal strength of the IVD are divided into five types. Clinical treatment of IDD is mostly involved decompression, fusion, and stabilization [12], and currently, there is no effective means to interfere with the degeneration process. The focus of studies on IDD is relatively fragmented, involving only limited proteins and factors, and cannot be used to systematically understand metabolism of related substances and their interaction mechanisms. The discovery of metabolomics in the 21st century [13] enables new understanding of a variety of biological functions. The easy-to-detect and easily identifiable properties of metabolites result in an effective bridge between molecular biology research and macroscopic cell behavior [14–16].

This study used mass spectrometry and nuclear magnetic resonance (NMR) metabolomics to explore metabolic characteristics of each process of IDD to understand the biological characteristics of the IVD. In addition, the findings of this study show the degeneration process from the level of material metabolism. The findings were verified through omics imaging, molecular biology, and histopathology thus establishing a complete metabolic profile. These findings showed the degenerative mechanism of antioxidation attenuation of the IVD.

## 2. Materials and Methods

### 2.1. Laboratory Animals and Ethics Statement

48 healthy Sprague-Dawley (SD) rats were purchased from Shandong Jinan Animal Experiment and Breeding Center. The weight of animals ranged between 557 ± 31.5 g for males and 509 ± 26.3 g for females. Male rats were raised in different cages from female rats. Animals were maintained under suitable temperature and light control conditions (22 ± 2°C, 12 h light-dark cycle) and provided with rodent food and water *ad libitum*. All experimental procedures were approved by the Ethics Committee of Qingdao University (approval number: QDFY-19-012-03) and were implemented in accordance with relevant guidelines and regulations.

### 2.2. Handling and Grouping of Experimental Animals

Twenty-four 6-month-old healthy SD rats of the same strain were selected and were similar in size and weight. Three consecutive segments of intervertebral space were selected for ultrasound-guided puncture (the segment was Co3-6), with no intervention for grade I IVD, unidirectional puncture with a fine puncture needle for grade II intervertebral disk, and multidirectional puncture with a thick puncture needle for grade III intervertebral disk. Three weeks later, 3.0 T magnetic resonance imaging (MRI) T_2_WI sequence and computed tomography (CT) scan confirmed establishment of the model.

### 2.3. Sources and Processing Methods of Human Samples

Selection criteria for experimental subjects were as follows: participants who presented with degenerative disease or acute injury of the lumbar spine that required IVD ectomy or discectomy and MRI can be performed at the corresponding time node before and after surgery were included in this study. Patients with tumors, infections, malformations, immune diseases, genetic diseases, metabolic diseases, and the young (<16 years) and elderly (>80 years) were excluded from the study. *In vitro* samples were obtained from 16- to 35-year-old patients with acute trauma. In this study, *in vivo* and *in vitro* experiments were performed after obtaining informed consent of patients and their families. All participants met ethical standards.

### 2.4. Data and Sample Acquisition Method

Image data was obtained by GE®3.0 T for preoperative MRI T_1_WI, SAG-FSE-TSE, T_2_WI, diffusion tensor imaging (DTI) and magnetic resonance spectroscopy (MRS). The corresponding relationship database of image data was established using patient information. Samples were collected by conventional surgical methods within 24 hours of injury or onset based on the preoperative MRI lesion responsibility segment. Samples were divided into four parts (for mass spectrometry, MRI, histopathology, cell culture, and molecular biology detection) and washed with distilled water. Samples were stored in vacuum packages and stored at low temperature.

### 2.5. Histopathological and Cellular Experiments

Paraffin-embedded sections: the attached tissue was separated from the spine, and tissues sections were fixed with 4% paraformaldehyde (Dalian Meilun Biological Co., Ltd., China) for 72 h. EDTA (Solarbio, China), NaOH (Shanghai Abel Chemical Company, China), and HCL (Tianjin Damao Reagent Company, China) were used to produce decalcification agent (pH 7.3-7.4) for decalcification. The decalcification agent was changed every week, for 4 weeks. After decalcification, paraffin-embedded sections of decalcified tissue (thickness 3-4 *μ*m) were obtained.

#### 2.5.1. HE Staining

To explore the tissue structure, the paraffin sections were stained using the HE kit (Solarbio). Paraffin sections were deparaffinized with xylene (Dalian Meilun Biological Co., Ltd., China) and washed with gradient ethanol (Dalian Meilun Biological Co., Ltd., China) and then rinsed with distilled water for hydration. Tissue sections were then stained with hematoxylin staining solution, differentiation solution, and eosin staining solution. After staining, gradient ethanol was used for dehydration, and then, tissues were treated with xylene : carbolic acid (3 : 1) to make them transparent and then sealed with neutral resin. Tissue sections were then observed under a light microscope.

#### 2.5.2. Masson Staining

To explore distribution and content of collagen fibers in tissues, paraffinized sections were stained with the Masson kit (Solarbio). In summary, paraffin sections were deparaffinized with xylene (Dalian Meilun Biological Co., Ltd., China) and stained with Weigert iron hematoxylin. Fluid staining was used for acidic differentiation. Further, tissues were stained with Ponceau-magenta staining solution. Tissue sections were then washed with weak acid working solution and phosphomolybdic acid solution. Tissues were stained with aniline blue staining solution, rinsed with weak acid working solution, and dehydrated with 95% ethanol (Dalian Meilun Biological Co., Ltd., China). Xylene solution was added to make tissues transparent, and tissues were then sealed with neutral resin and observed under a light microscope.

#### 2.5.3. Safranin O-Fast Green Staining

To assess the relative distribution of bone and cartilage in the tissue, paraffinized sections were stained with the Safranin O-Fast Green Kit (Solarbio). Paraffin sections were deparaffinized with xylene (Dalian Meilun Biological Co., Ltd., China). Weigert iron hematoxylin staining solution was then used for staining and acidic differentiation solution for differentiation. Fast green dye solution was used for dip dyeing, and tissues were washed with weak acid working solution. Tissues were dip-stained with Safranin O and dehydrated with gradient ethanol (Dalian Meilun Biological Co., Ltd., China). Xylene was used to make tissues transparent, and the film was mounted with neutral resin. Tissues were then observed under a light microscope.

#### 2.5.4. Immunohistochemistry

Immunohistochemistry was used to evaluate the expression levels of MMP3/TIMP1 in tissues. Paraffin wax slices were baked overnight. Xylene (Dalian Meilun Biological Co., Ltd., China) was used for dewaxing, washed with gradient ethanol (Dalian Meilun Biological Co., Ltd., China), and TBST buffer was used for hydration. 3% hydrogen peroxide solution was added for blocking, and tissue sections were then washed with TBST solution. The slices were washed with TBST solution after antigen retrieval. Tissue slices were placed flat in the wet box, and the tissue boundary indicated with a hydrophobic pen, and then, 3% BSA solution was added. The blocking solution was aspirated, and primary antibody was added (MMP3/TIMP1 antibody purchased from Santa Cruz), and then, the mixture was incubated overnight at 4°C. Sections were warmed to room temperature and washed with TBST solution. Tissue sections were then incubated with secondary antibody at room temperature, and color was developed with diaminobenzidine (DAB) solution, under controlled reaction time. Tissues were then observed under a microscope. Hematoxylin solution was used for dyeing and counterstaining, and then, tissues were alkalized with alkalized water. Tissue sections were dehydrated with gradient ethanol (Dalian Meilun Biological Co., Ltd., China), and xylene was used to make sections transparent. The slide was mounted with neutral resin, and then, tissue sections were observed under a light microscope.

#### 2.5.5. Cell Morphology and Cell Activity

AF and EP were separated from isolated IVD tissues. Primary NP cells were extracted from the NP tissue using the type II collagenase method. Cell culture flask containing NP cells was placed at 37°C, under 5% CO_2_ incubator. The flask was taken out on days 1, 3, 5, and 7 for observation of cell morphology. The CCK-8 method was used to quantitatively analyze proliferation activity of NP cells at various levels. Cells were counted and seeded in a 96-well plate. Cells were then incubated in a 5% CO_2_ incubator at 37°C, for 4 hours; 10% CCK-8 was added by changing the medium. After addition of CCK-8, cells were incubated for 2 h in the incubator, and the absorbance was determined at 450 nm using a microplate reader.

### 2.6. Western Blot (WB) Analysis

Western blot was used to detect degenerative factors in IVD samples. In summary, separation gel and concentrated gel were prepared, and samples were loaded according to the measured protein concentration. The application antibody product number information is TIMP1 (bs-0415R), TIMP2 (10396-H01H), TIMP3 (ab159704), MMP1 (bs-4597), MMP9 (sc-13520), MMP13 (310471-T40), MATN3 (ab106388), IL11 (ab76589), Aggrecan (bs-11655R), Collagen II (bs-11929R), and *β*-actin (bs-0061R). Samples were subjected at constant voltage 80 V electrophoresis. After entry of bromophenol blue dye in the separation gel, the constant voltage was changed to 100 V and electrophoresis was performed until the bromophenol blue dye reached the bottom of the separation gel. After cutting the glue, the membrane was transferred in a transfer box. PVDF membrane was sealed with skimmed milk powder, and primary antibody was added at 4°C overnight. After washing samples in the TBST shaker, secondary antibody was added to samples and incubated for 1 h. Liquid A and liquid B in the ECL kit were used to prepare a luminescent liquid 1 : 1. The liquid was added to the PVDF membrane and then placed on the Bio-Rad developing instrument for development. Expression of the target protein was observed, and images were obtained for analysis.

### 2.7. Routine Sequence Scanning and Pfirrmann Classification

A 3.0 T MRI machine (GE®-MR750, USA) was used to perform one-time detection of T_1_WI, T_2_WI, and other sequences after setting the relevant scanning parameters. Pfirrmann grading of the IVD was determined based on T_2_WI.

### 2.8. DTI Detection Scan and ADC Value and FA Value Analysis

A 3.0 T MRI machine (GE®-MR750, USA) magnetic resonance imager was used to perform DWI imaging. Data were imported into PACS system to determine apparent diffusion coefficient (ADC) and partial anisotropy index (fractional anisotropy (FA)) of region of interest (ROI) related to the intervertebral disc nucleus pulposus.

### 2.9. NMR *In Vivo*^1^H Spectrum Detection Scan and Data Processing

GE®3.0 T DISCOVERY MR750 was used to obtain the original data and which was then preprocessed with jMRUI_v6.0 beta to obtain MRS fitting spectrum. Peak calibration was performed on the final curve to obtain relevant compound information. Analysis of ^1^H spectrum was mainly the H atom signal distribution of characteristic groups. Distribution of the compound represented by each peak was assigned a compound name based on the identification of synchronous mass spectrometry. The assignment was determined by standard compound NMR spectrum database (https://sdbs.db.aist.go.jp/sdbs/cgi-bin/cre_index.cgi?+lang=eng). The assignment indicated the ^1^H spectrum representative peaks of each level of IVD.

### 2.10. Nontargeted Metabonomics Analysis by High-Resolution Mass Spectrometry (HRMS)

Ultrahigh-resolution performance liquid chromatography-Q-HF-X hybrid-high-resolution mass spectrometer detection (UHPLC-Q-HFX-HRMS): samples were washed and dried and then pretreated, extracted, and homogenized with a homogenizer and then sampled for testing. Mass spectrum platform was used for analysis of samples. Total ion chromatogram (TIC) was obtained from the raw data after noise reduction, baseline correction, peak alignment, data binning, peak standardization, and standardization.

Metabolite difference analysis: multivariate statistical analysis was performed using principal component analysis (PCA) and orthogonal partial least squares discriminant analysis (OPLS-DA) to identify labeled metabolites, and heat maps were generated. Permutation test and S-plot scatter plot were used to analyze significance of metabolite differences.

Metabolic pathway analysis and calibration: functional analysis was performed based on the differential metabolites. Related metabolic pathways were identified through enrichment analysis and topological structure. The five-level IVD groups were compared to draw statistical charts.

### 2.11. Bioinformatics Analysis

The microarray dataset of IDD was retrieved from the NCBI GEO database. Metabolic pathways of differentially expressed proteins were analyzed. Machine learning algorithms were used to further screen and determine the metabolic pathways of IDD. The ssGSEA tool was used to analyze the different subgroups of RNA-seq data. The proportion of each metabolic pathway was determined, and the correlation between metabolic pathways and specific genes was analyzed.

The ssGSEA tool was used to analyze RNA-seq data of different subgroups of patients to infer the relative proportions of 43 metabolic pathways. The “pheatmap” package was used to generate a heat map of metabolic pathways and compare distribution of metabolic pathways between the two groups. The “vioplot” package was used to determine the relative content of metabolic pathways, and the “corrplot” package was used to determine correlation coefficients between metabolic pathways and specific genes. *P* < 0.05 was considered as statistically significant.

### 2.12. NMR *In Vitro*^1^H Spectrum Sample Collection and Data Processing

NP tissue was lysed with RIPA and then ground. Tissue sample was resuspended in dichloromethane and methanol and then homogenized. Original data was obtained by computational analysis. Preprocessed data was based on the iconic lactate peak. The spectrum was aligned, and the full spectrum was labeled and integrated to obtain the full spectrum peak data and peak shape distribution. After identification of the coupling situation of each peak, substance integration quantitative analysis was performed to obtain a compound prediction list.

### 2.13. Analysis of TOCSY Two-Dimensional NMR Spectra *In Vitro*

The “two-dimensional correlation spectrum” experiment was used to label the IVD qualitative IVD marker compounds. The original data was processed by Mestrenova 14.0.0 through spectral baseline correction, *f*1, *f*2 phase correction, Fourier transform, window function, and zero function adjustment and signal suppression. After processing, the lactic acid (Lac) peak (*f*1 = 1.35 ppm, *f*2 = 4.16 ppm) was calibrated and a total correlation spectroscopy (TOCSY) spectrum generated. The newly identified compounds represented by each peak were then identified and assigned.

### 2.14. Statistical Analysis

Data obtained were analyzed based on their respective schemes and methods for intragroup difference statistics and between-group difference statistics. Quantitative data were represented as *X* ± *S* and parametric testing was done using a *t*-test. Nonparametric was represented as median and interquartile plotting rank sum test was used for nonparametric data. Multigroup analysis of quantitative data was performed using one-way analysis of variance followed by Kruskal-Wallis test. Correlation analysis was performed to compare expression in the different groups. *P* < 0.05 was considered as statistically significant.

## 3. Results

### 3.1. Establishment of the Rat IDD Model

**Figure 1:**
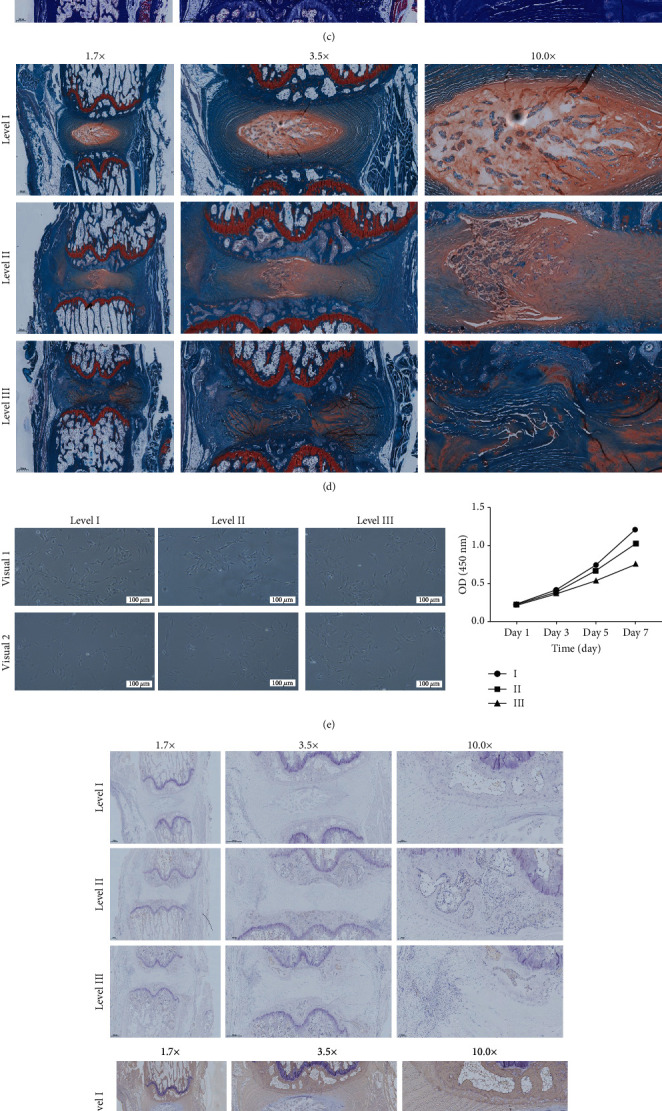
(a) MRI T_2_WI imaging and CT scan imaging 3 weeks after inducing the puncture. (b) HE staining image of sagittal section. (c) Masson staining image of sagittal section. (d) Safranin O-fast green staining of sagittal section. (e) Light microscope image ① and cell growth line chart ② showing the morphological characteristics. (f) Immunohistochemistry of MMP1 ① and TIMP1 ②. (g) Western blot strip ① and grayscale images ② of TIMP1, MMP1, Aggrecan, and Collagen II.

Crawling posture of rats and the locking structure of the spinal joints hindered formation of the natural degeneration of the IVD. Disc puncture was performed to simulate natural degeneration. The principle was to destroy distribution of NP cells and the normal structure of the IVD. Three weeks after the puncture, MRI T_2_WI imaging showed that the model met the preset imaging conditions. In addition, CT showed significant differences in the height of the intervertebral space between models of different levels (Figure 1(a)).

Histopathological analysis showed significant differences in the macroscopic structure of the NP and AF and the relative microscopic morphology, coloring, and distribution of NP cells in the three-level IVDs in terms (Figure 1(b)). Furthermore, differences in morphology and distribution of collagen fibers and cartilage tissue were observed in the three-level IVDs (Figures 1(c) and 1(d)). Cell morphology and adherent growth state were different in the three-level IVDs. Proliferation activity was quantified by CCK-8 (Figure 1(e)).

Matrix metalloproteinase-1 (MMP-1) stained deeper in the NP-like cell with IDD (Figure 1(f)), and its expression was increased (*F* = 884.1, *P* < 0.05). Tissue inhibitor of metalloproteinase 1 (TIMP1) was light brown in the NP tissue (Figure 1(f)). TIMP1 expression increased with the level of IVDs; however, a decrease in the ratio of TIMP1 expression to MMP1 expression was observed (*F* = 264.1, *P* < 0.05).

WB results showed a decrease in the expression of structural characteristic proteins Aggrecan and Collagen II, whereas the expression of TIMP1 and MMP1 proteins related to ECM metabolism was higher (Figure 1(g)). Notably, the expression of MMP1 was higher compared with that of TIMP1.

### 3.2. Metabolomics of the Rat IDD Model

**Figure 2:**
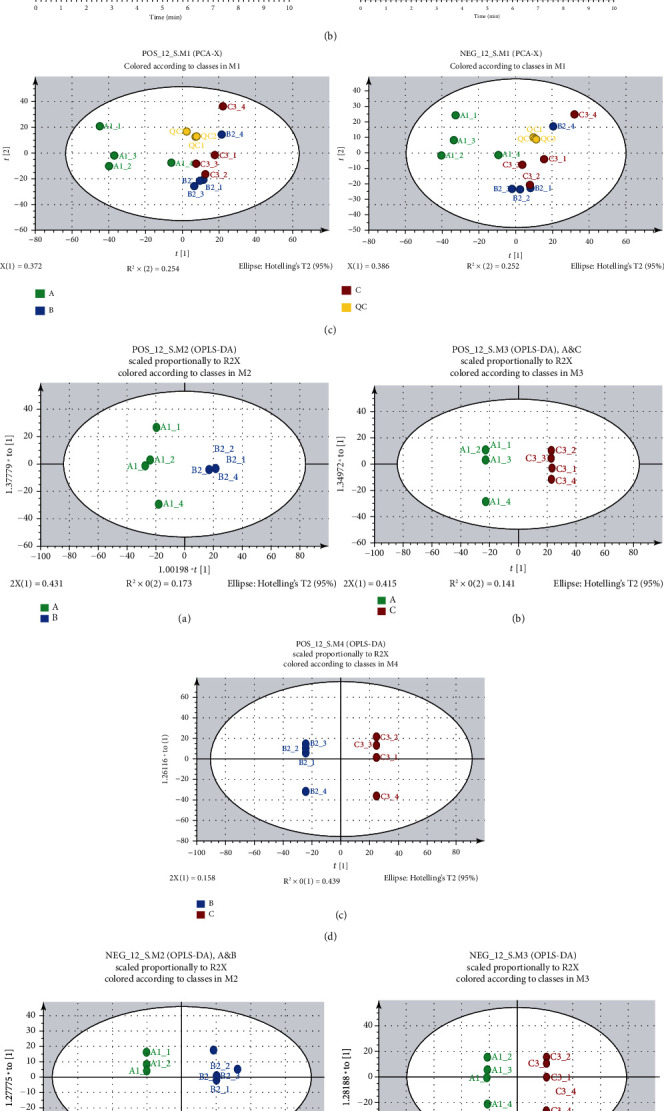
(a) Flowchart of metabolomics. (b) Total ion current (TIC) diagram. (c) Principal component analysis (PCA). QC: quality control group. (d) OPLS-DA diagram of each group in cation mode: (A) A/B group comparison, (B) A/C group comparison, and (C) B/C group comparison. (e) OPLS-DA diagram of each group in anion mode: (A) A/B group comparison, (B) A/C group comparison, (C) B/C group comparison.

UHPLC-Q-HFX-HRMS was used to perform mass spectrometry metabolic analysis on rat IVD samples of different levels (Figure 2(a)). The superimposed spectra of the three groups of samples formed an overall picture of metabolites (Figure 2(b)). The total number of peaks in the cation mode of HRMS was 4505. Notably, 313 effective metabolites were identified. The total number of peaks in the anion mode was 4430, resulting in identification of 226 metabolites (Figures 2(d) and 2(e)).

PCA was used to perform dimensionality reduction processing of data features. Analysis showed that the distribution ranges of groups B and C in PCA anion and cation mode were mostly overlapping, whereas the areas of group B and C were separated from the area of group A (Figure 2(c)). This finding implies that group A had unique independence, whereas groups B and C showed homogeneity in the metabolic data characteristics. OPLS-DA model operation was used to further distinguish the differences between groups to determine the sample attribution thus determining the relationship between disc grading and metabolite expression. OPLS-DA model under the anion and cation mode showed differences in the three groups. However, significance of the differences needs further exploration. Permutation test was performed on OPLS-DA pairwise comparison model, and covariance and correlation coefficient of each variable were used to generate an S-plot. Analysis showed no significant difference between groups B and C.

### 3.3. Metabolic Pathway and Metabolite Analysis

**Figure 3:**
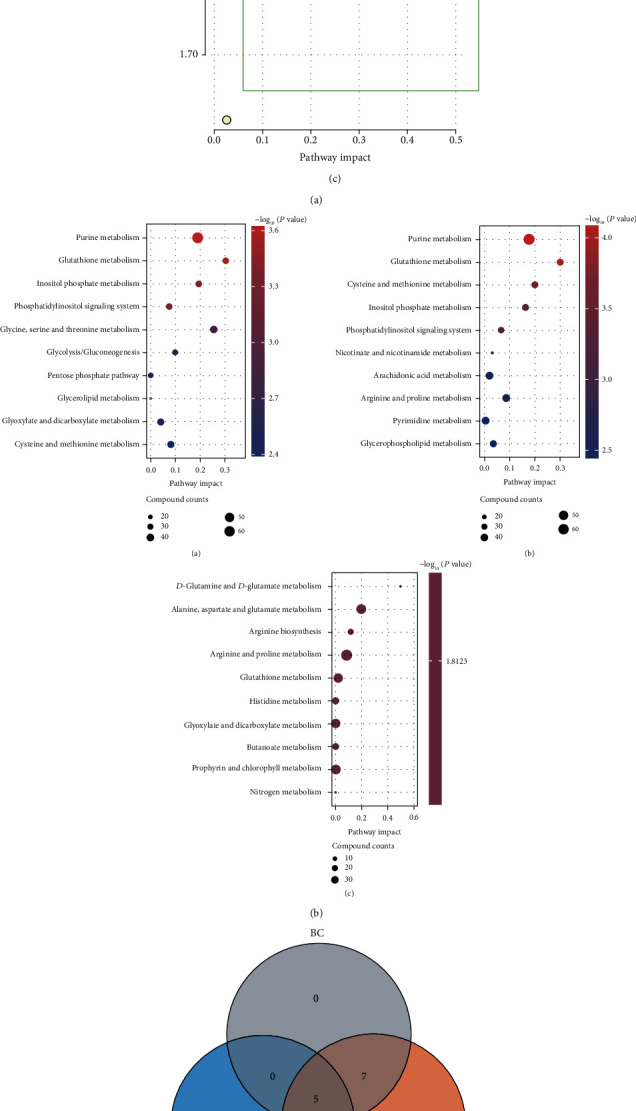
(a) Enrichment analysis of metabolic pathways in three groups of samples: (A) A&B group, (B) A&C group, and (C) B&C group. (b) Enrichment analysis of active metabolic pathways of three groups of samples: (A) A&B group, (B) A&C group, and (C) B&C group. (c) Venn diagram showing differences in metabolic pathways of the three groups of samples. (d) Metabolite volcano map of the three groups of samples, A&B group, A&C group.

Pathway enrichment analysis was performed using R software. A topological map of the metabolic pathway enrichment network of each comparison group was generated (Figure 3(a)). Metabolic pathways with significant differences were identified (Table 1). Main metabolic pathways before and after degeneration included ① glutathione metabolism, ② glycine, serine, threonine metabolism, ③ inositol phosphate metabolism, ④ purine metabolism, ⑤ amino sugar and nucleotide sugar metabolism, ⑥ glycolysis/gluconeogenesis, ⑦ cysteine, methionine metabolism, ⑧ pentose phosphate pathway, and ⑨ alanine, aspartic acid, glutamate metabolism. Enrichment analysis of the above metabolic pathways based on the degree of activity (Figure 3(b)) was similar to the enrichment of differential metabolic pathways, implying that the consistency of pathway enrichment models was good. To explore the core degree of each metabolic pathway, a Venn diagram of the metabolic pathways in the early and late stages of IDD was constructed (Figure 3(c)). Analysis showed overlapping of more metabolites between group AB and group AC whereas group BC showed fewer overlapping. Group AB and group AC were used to construct a volcano plot based on the degree of contribution (VIP) and metabolite analysis reliability (*P* value) (Figure 3(d)). The fold change was set at >2 times, *P* value < 0.05 to filter out differences in expressed metabolites. A total of 15 different metabolites were identified between group A and group B, which are implicated in 6 main metabolic pathways. A total of 19 different metabolites were identified between group A and group C, which are implicated in 6 main metabolic pathways.

### 3.4. Biological Information Database Comparison

**Figure 4:**
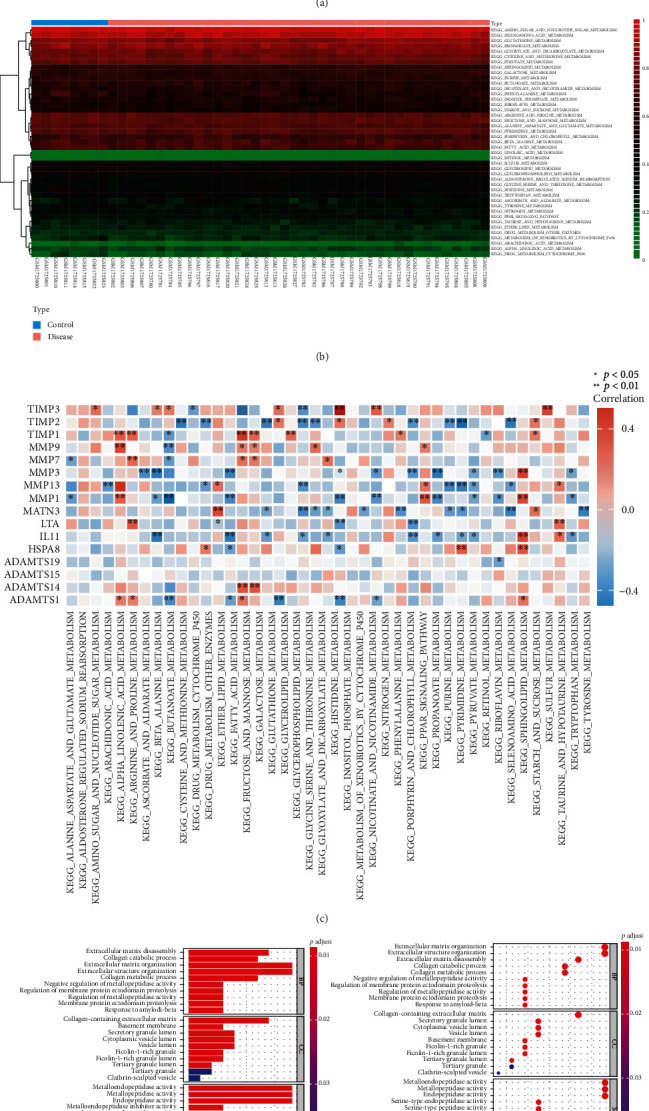
(a) Relative percentages of 43 metabolic pathway subgroups in 48 human samples. (b) Heat map of the expression of metabolic pathways differentially expressed in human IDD. (c) Heat map of the correlation between core genes and metabolic pathways, red color shows positive correlation and blue color shows negative correlation; ^∗^*P* < 0.05 and ^∗∗^*P* < 0.01. (d) GO enrichment analysis bar scale chart, GO enrichment analysis load bubble chart.

A total of 43 metabolic pathway subgroups in the 48 human samples of the GSE70362 dataset were retrieved from the NCBI GEO bioinformation database. The expression of the 43 metabolic pathways was analyzed and summarized (Figure 4(a)). Functional enrichment analysis was used to compare the 43 differentially expressed metabolic pathways. A heat map was generated to show the expression of 43 metabolic pathways (Figure 4(b)). Analysis showed that the expressions of human and rat IVD material metabolism pathways were similar, and the metabolism mode was similar. The “corrplot” package was used to analyze the correlation coefficients of metabolic pathways and specific genes. Metabolites in each pathway were analyzed, and the core factors with significant differences were identified as the significant factors of the pathway (Figure 4(c)). Gene Ontology (GO) function enrichment analysis was performed to explore the functions of metabolites in terms of the three biological characteristics of biological pathways: biological process (BP), cellular component (CC), and molecular function (MF) positioning and cellular processes. Analysis showed significant changes in the ratio of biological process including collagen catabolism, ECM synthesis, ECM decomposition, and collagen metabolism (Figure 4(d)). Analysis of the contribution degree of each path showed that the decomposition of collagen and ECM was the main source of the difference in IDD. This finding implies that cell function of ECM collagen and matrix membrane was mainly affected.

### 3.5. Mass Spectrometry Analysis of Human Samples

**Figure 5:**
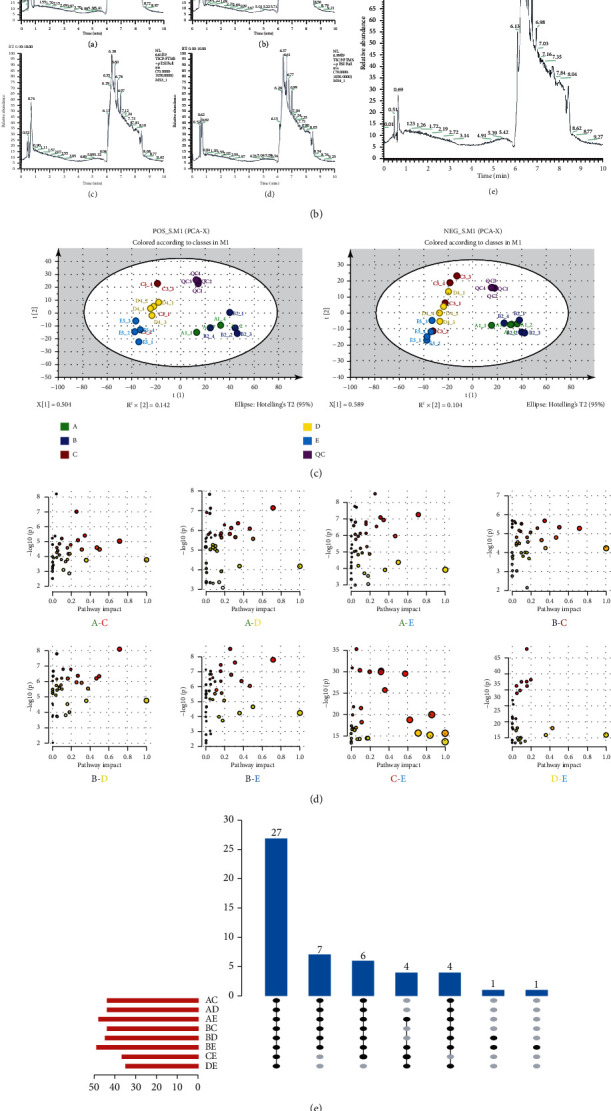
(a) Mass spectrometry (UHPLC-Q-HFX HRMS) metabolomics flowchart. (b) Total ion current (TIC) diagram of human IVD. (c) Principal component analysis (PCA). (d) Differential metabolic pathway enrichment among groups. (e) Upset diagram of metabolic pathways of different substances in each group. (f) Western blot strip and grayscale map of degeneration factors related to key pathways ① glycine-serine-threonine, ② galactose, and ③ structural protein.

IVD of patients was analyzed by mass spectrometry metabolomics (Figure 5(a)). IVD was divided into five grades based on Pfirrmann classification. Information of isolated tissues that were screened and submitted is shown presented in Table 2. The TIC of substances obtained from analysis of the IVD samples of each level is shown in Figure 5(b). PCA showed that the samples of groups A and B were relatively close, and those of group C and group D were relatively similar (Figure 5(c)). Analysis using OPLS-DA model, permutation test, and S-plot load diagram showed that the five levels of human IVD can be divided into three metabolic stages.

Metabolic pathway enrichment analysis showed significant differences in metabolic pathways in the A-E group (Figure 5(d)). Notably, 11 types of amino acid metabolism pathways accounted for 36.7%, and 6 types of sugar metabolism pathways accounted for 20% of the total pathways. These findings show that amino acid and sugar metabolism were dominant. Furthermore, the most important common differential metabolic pathways between groups A and B and groups C and D included ① amino acid metabolism pathways: metabolism of glycine, serine, and threonine, metabolism of cysteine and methionine, acid biosynthesis, metabolism of arginine and proline; ② carbohydrate metabolism pathway: glycolysis/gluconeogenesis, galactose metabolism; and ③ small molecule, lipid metabolism pathway: taurine and hypotaurine metabolism, pyruvate metabolism, and inositol phosphate metabolism (Figure 5(e)). Further, the most important common differential metabolic pathways between groups C, D, and E included ① amino acid metabolism pathway: arginine and proline metabolism, glycine, serine, and threonine metabolism; ② sugar metabolism pathway: fructose and mannose metabolism; ③ small molecule and lipid metabolism pathway: inositol phosphate metabolism; and ④ nucleotide metabolism pathway: pyrimidine metabolism, purine metabolism, amino sugar, and nucleotide sugar metabolism.

Analysis of degeneration-related factors of these key pathways showed that metabolism of glycine-serine and threonine (MMP13, MATN3, TIMP2, and TIMP3) after the degeneration decreased expression of related molecules compared with the levels before degeneration (Figure 5(f)). In addition, metabolism in the late degeneration axis expression was significantly low. Analysis of galactose metabolism (ADAMTS1, TIMP1, MMP7, and MMP9) showed that ADAMTS1 reached its peak in grade II IVDs. Moreover, change in expression level of TIMP1, MMP7, and MMP9 was consistent with decomposition of ECM of IVDs. Expression levels of structural proteins (Collagen II and Aggrecan) decreased progressively throughout the degeneration process.

### 3.6. NMR Analysis of Human Samples

**Figure 6:**
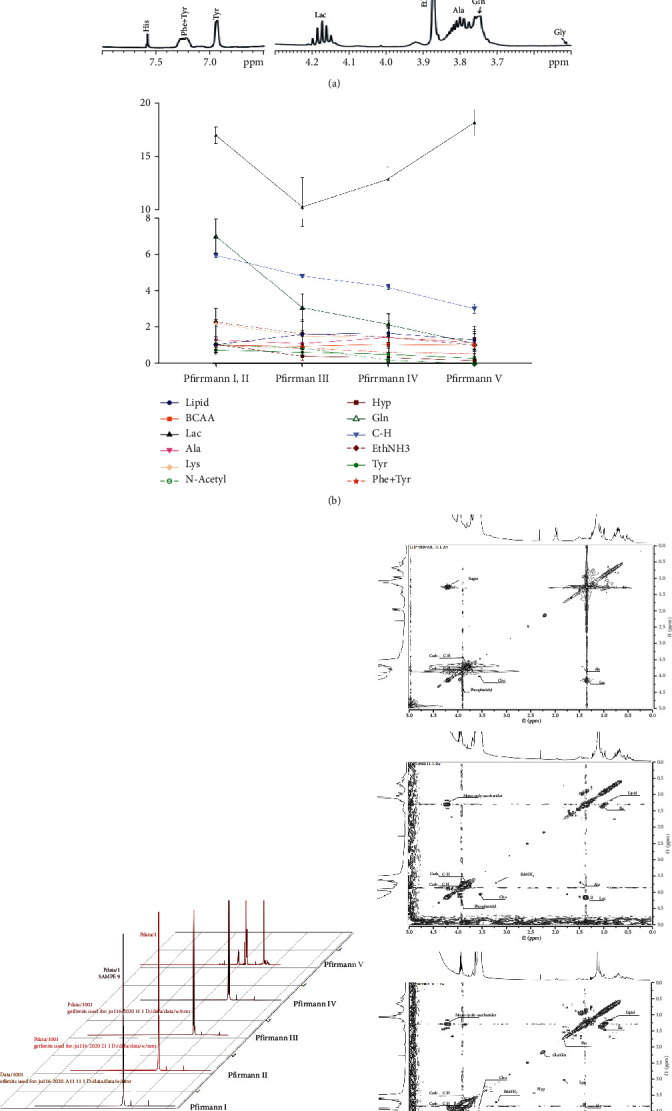
(a) Pfirrmann V grade disc NMR ^1^H spectrum assignment. (b) Quantitative analysis chart of NMR ^1^H spectrum of Pfirrmann I~V IVD. (c) Pfirrmann I~V grade IVD NMR ^1^H spectrum peak line change arrangement diagram. (d) Two-dimensional TOCSY spectrum of Pfirrmann I ①, III ②, and V ③ disc.

Distribution of compounds represented by each peak was identified through the attribution based on synchronous mass spectrometry identification. Representative peaks of the ^1^H spectrum of each level of IVD were identified based on the attribution and data from the standard compound NMR spectrum database. The V-level disc was used as a representative sample (Figure 6(a)). Designated peaks of the ^1^H spectrum of each graded IVD were manually integrated to determine the levels of metabolites for each peak. This was performed to determine the concentration of the substance in the unit magnetic field volume in the solution, and integral value of each substance was recorded and trend chart generated (Figure 6(b)). Notably, substances were not included in the statistics. Arrangement of the peaks of each spectrum is presented in Figure 6(c). One-dimensional ^1^H spectroscopy produces a large amount of H signal overlap in the NMR system. Although it is used to accurately identify the types of amino acid residues, it is challenging to identify the spatial conformation of biopolymers such as proteins and proteoglycans. The “two-dimensional correlation spectrum” experiment was used to further determine types of polymer compounds by comparing with the clinical MRS (Figure 6(d)).

### 3.7. Verification of Human Imaging

**Figure 7:**
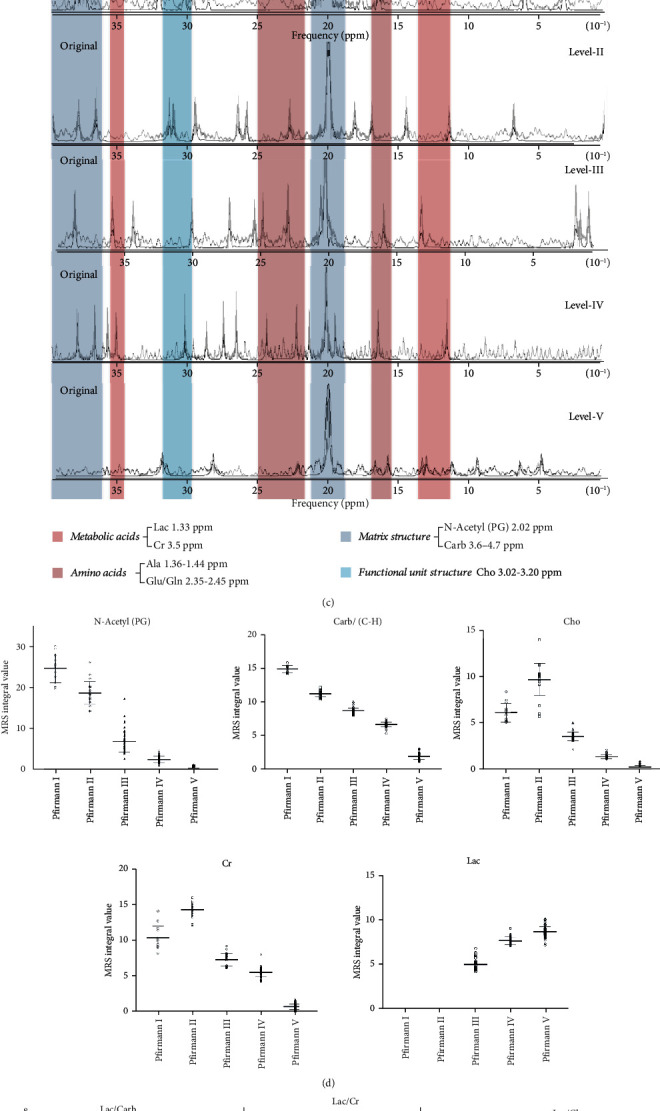
(a) DWI, ADC, and FA axis diagram of each grade of IVD. (b) Analysis of ADC value and FA value of Pfirrmann I-V IVD DTI imaging. (c) Identification and qualitative induction of MRS measurement peaks of Pfirrmann I-V IVDs *in vivo*. (d) Quantitative value statistics of five single peaks of MRS in each level of IVD. (e) Trend change graph of the integral value ratio of ① lactate, ② choline compound, and ③ N-acetyl resonance compound.

Analysis of DTI imaging axial map of each level of the IVD showed that Pfirrmann I-V level represents gradual decrease of the high signal of water molecule activity (Figure 7(a)). ADC value and FA value of the IVD NP area were determined in the five levels (Figure 7(b)). Analysis showed that the ADC value of grade I-IV IVDs gradually decreased with degeneration of the disc (*F* = 161.0, *P* < 0.01). Notably, grade V IVDs were not detectable in DTI imaging. In grade V, ADC and FA values were not obtained, implying that the measured value range exceeded the high limit or cannot be imaged. This finding shows that the ADC value of degenerative IVDs gradually decreased, whereas the FA value increased with degeneration. Notably, analysis showed significant differences in ADC and FA values between groups (*F* = 3810.0, *P* < 0.01).

Pfirrmann grade IV disc spectrum peak identification was similar, from low field to high field (Figure 7(c)). The values are as follows: lactic acid (Lac) 1.33 ppm, alanine and propionate compounds (Ala) 1.36-1.64 ppm, N-acetyl resonance compound or collagen compound (N-Acetyl/PG) 2.02 ppm, glutamate and glutamine compound (Glu/Gln) 2.35 ppm-2.45 ppm, total choline compound (Cho) 3.02 ppm-3.20 ppm, muscle acid (Cr) 3.5 ppm, carbohydrates, and hydrocarbons and lipids (Carb) 3.6 ppm-4.7 ppm. The overall trend of levels of metabolites decreased with increase in degeneration intensity. Levels of metabolites in the grade V IVD were significantly low compared with other groups. N-Acetyl, Cho, Lac, Cr, Carb, N-Acetyl/Carb, N-Acetyl/Cr, Carb/Cr, N-Acetyl/Cho, and Carb/Cho were set based on peak imaging characteristics (Figure 7(d)). Absolute quantification of each value does not necessarily have a good trend under the conditions of NMR spectroscopy; therefore, the ratio of the integral value of each single peak was used to determine quantity of the spectrum material (Figure 7(e)).

## 4. Discussion

Degeneration is a multistage physiological process with complex factors. IVDs at different degeneration stages are characterized by differences in morphology, pathology, and cell biology [17]. Clinically, IDD is divided into pre-, early, and late-stage degeneration [18]. The gold standard of IDD in the imaging field is Pfirrmann classification [17]. Microscopic analysis shows that many proteins and related degenerative factors are implicated in the degeneration process [19–22]. Interaction of these components further deteriorates degeneration. The three levels of degeneration process are relatively independent from each other. Advances in metabolomics provide a means to establish a systematic degeneration mode. In this study, rats and degenerative disc disease patients with high gene homology were used for metabonomics analysis. The overall picture of IDD was determined through imaging, histopathological analysis, and molecular biology methods.

Currently, MRI is the main method for noninvasive diagnosis of IDD [23]. Its principle is to collect proton relaxation signals in the body and construct a digital matrix to describe the spatial distribution characteristics of sample nuclei. MRI is used to visually observe morphological changes of the tissue structure [24]; however, due to the inevitable loss of the original characteristics of the information during the signal conversion process, it is challenging to generate the accurate profile within the tissue. Therefore, imaging is only useful for “appearance” stage. Advances in metabolomics allow immediate internal display of the organization of tissues [25]; however, its process is tedious and costly [26]; therefore, it is not widely used. This study used NMR metabolomics combined with mass spectrometry metabolomics to explore the metabolic profile of IVD and further performed quantitative and qualitative analysis of spectrograms to establish an *in vivo* metabolism model of noninvasive detection of degeneration.

Analysis showed that the main metabolic processes were carbohydrate and protein metabolism, mainly involving matrix components such as collagen and proteoglycans. Therefore, this study focuses on amino acid metabolism and sugar metabolism. Analysis of TIC, OPLS-DA, and the corresponding permutation test and S-PLOT load diagram showed that group A without intervention in the overall metabolic pattern of rat IVDs was significantly different from other groups. On the contrary, groups B and C showed similar trends in expression of metabolites. Therefore, the two stages before and after degeneration were explored.

The main different metabolic pathways before and after degeneration were mainly related to three types of biological activities including ① carbohydrate utilization (amino sugar and nucleotide sugar metabolism, pentose phosphate pathway, alanine metabolism, and glycolysis/gluconeogenesis pathway), ② antioxidant pathways (glutathione metabolism, cysteine, and methionine metabolism), and ③ structural protein synthesis and degradation (Gly-Ser-Thr metabolism) [27, 28]. Notably, most of the pathways revolved around the Gly-Ser-Thr metabolism axis [29, 30]. The Venn diagram of differential metabolic pathways showed that the metabolism of Gly-Ser-Thr was different in intersection of AB and intersection of AC. This was attributed by the significant changes in the functional status of the Gly-Ser-Thr axis before and after degeneration [31].

Analysis of the difference in metabolites showed that marker metabolites in the predegeneration stage were mainly implicated in carbohydrate metabolism pathways including pentose phosphate pathway, glycolysis/xenogenesis, and amino sugar and nucleotide sugar metabolism and protein metabolism pathways including serine and threonine metabolism, cysteine and methionine metabolism, and small molecule metabolism mainly inositol phosphate metabolism. In addition, marker metabolites in the late stage of degeneration were mainly involved in sugar metabolism pathways such as amino sugar and nucleotide sugar metabolism, proteoglycan structural metabolism, and tricarboxylic acid (TCA) cycle; protein metabolism pathways such as cysteine and methionine metabolism and serine and threonine metabolism; and small molecule metabolism including niacin and nicotinamide metabolism and PGI2 metabolism. Consistent with the findings on metabolic pathways, the main biological processes involved in rat IDD were energy metabolism [32], biological oxidation [33], and structural protein synthesis and decomposition [34]. Gly-Ser-Thr metabolic pathways are involved in biotransformation and metabolic activities during degeneration [35].

Cysteine plays a role regulating glutathione synthesis. Glutathione is an important antioxidant and oxygen free radical scavenger in the body. Methionine is involved in destruction of membrane lipids produced by oxidative free radicals through multiple channels of oxidation thus protecting membrane-containing structures such as cells and mitochondria [36]. Downregulation of cysteine and methionine metabolism during degeneration process leads to decline of the antioxidant function in the internal environment of the IVD [37]. Peroxidation of tissue membranes promotes degeneration of the IVD. Amino sugars and nucleotide sugars play important role in the process of carbohydrate conversion and energy utilization [38]. Notably, amino sugars and nucleotide sugars were downregulated during the degeneration process. Pentose phosphate pathway and glycolysis are involved in supply of energy and reducing equivalents important for antioxidant activity. These changes during degeneration indicate a transition from multimode coexistence of energy utilization in the IVD to a single mode of proteoglycan structural metabolism, implying that material disintegration of the NP tissue occurred in the later stage of degeneration. Furthermore, decline of the overall function of the energy utilization system may be the initiating factor leading to accumulation of Lac in the late stage of IDD. TCA cycle is the final metabolic pathway and metabolic hub of the three major nutrients [39]. Downregulation of TCA cycle in the later stage of degeneration implies that the metabolites of the IVD are depleted, which is attributed to apoptosis of NP cells and the decline in their secretory function. Further, the Gly-Ser-Thr metabolic axis was downregulated in the early and late stages of degeneration [35], implying that the functional state of the metabolic axis is negatively correlated with the degree of degeneration. It is speculated that antioxidants are produced through the Gly-Ser-Thr metabolic axis. The Gly-Ser-Thr metabolic axis may play a role in delaying degeneration of the IVD through regulation of carbohydrate conversion and energy utilization and ultimately through formation of antioxidant agents.

Although the rat genome is highly homologous to human genome and the metabolic pathways are similar, it cannot be inferred that the metabolic profiles of the two organisms are the same. Therefore, human metabolic pathway subgroups were retrieved from the NCBI GEO database and used for induction. A heat map on the correlation between core genes and metabolic pathways showed that main changes are changes in the metalloproteinase families [40, 41] and their tissue-specific inhibitory enzyme family-related factors. Moreover, Gene Ontology functional enrichment analysis showed that decomposition of collagen and ECM is the main source of disc degeneration differences [19, 42, 43]. Further analysis on key pathway degeneration-related factors showed that expression of metabolites involved in the Gly-Ser-Thr metabolic pathways [35] (MMP13, MATN3, TIMP2, and TIMP3, IL-11) decreased after degeneration compared with before degeneration (I, level II ⟶ level III). Notably, expression of metabolites involved in this metabolic axis was extremely low in the late stages of degeneration (levels IV, V). These findings show that the metabolic axis is consistent with the degeneration process, and its function declines with increase in degeneration progresses. IL-11 is an important factor of fibrosis [44], and high expression level causes fibrosis of NP tissue [45]. Metabolites involved in galactose metabolism included ADAMTS1, TIMP1, MMP7, and MMP9. ADAMTS1 participates in biological processes by hydrolyzing proteoglycans [46] and inhibiting angiogenesis [47]. It reaches its peak at level II discs and initiates proteoglycan production. The process of resolution and decline in expression in the later stage may be related to the vascular growth in the IVD in the late stage of degeneration. In addition, changes in levels of TIMP1, MMP7, and MMP9 [48] were consistent with increased decomposition of the ECM of the IVD, resulting in progressive decline of structural proteins (Collagen II and Aggrecan) throughout the degeneration process. Molecular biology analysis shows the stable relationship between macrobiological processes and metabolomics. The relationship between macrobiological processes and metabolomics provides an avenue to verify the phased expression trend of metabolic pathways.

NMR is an effective tool for mass spectrometry metabolomics due to its universality and high throughput for hydrogen-containing metabolites [49]. Advantages of the quantification function of NMR allow quantitative analysis of sample metabolites at various stages [50]. NMR ^1^H spectrum identification and quantitative analysis showed that consistent with the mass spectrum metabolism findings, there was no significant change in the types of metabolites. Notably, Lac levels increased significantly with degeneration. Decrease in the level of phenylalanine, tyrosine, and other sugar metabolism regulating substances [51] with the degeneration of the IVD implied a decline in energy utilization efficiency and conversion of sugar utilization mode. Ethanolamine, EthNH_3_, is an intermediate metabolite of phospholipids [52], and its content decreases significantly with degeneration, implying that methionine has multiple pathways to prevent oxidation of membrane lipids by oxidative free radicals. Furthermore, levels of N-Acetyl decreased significantly with degeneration, indicating the process of dissolution of IVD structural substances. The presence of glycine residues in the final stage of degeneration indicates that the function of the Gly-Ser-Thr axis is effectively involved in the process of degeneration. However, ^1^H spectrum has poor specificity in identifying compounds. In order to separate effective compound components from the overlapping peaks and peaks with small amplitude, TOCSY two-dimensional spectrum was used to transfer the magnetization vector using the direct coupled spin method [53] thus allowing observation of the same spin. Analysis of relevant signals between the indirectly connected nuclei in the system showed that the existing form of sugars changes from various types of sugars to monosaccharides/polysaccharides. These changes indicate that the types of sugars available in the late stage of degeneration are reduced, and the corresponding metabolites result in reduction of carbohydrate metabolism pathways. In addition, glutamic acid/glutamine decreased in the initial stages then increased in the late stages of degeneration. Decrease in the early stage of degeneration results in inhibition of glutathione synthesis, which affects the antioxidant capacity of the Gly-Ser-Thr metabolism axis. On the other hand, the abnormal increase in the later period is related with accumulation of Lac. Increase in lactate is attributed to the decay process of the energy utilization system. In addition, increase in the types of amino acids in the other two-dimensional spectrum (such as alanine) indicated decomposition of the main structural proteins of the NP.

*In vitro* NMR ^1^H spectrum and the TOCSY two-dimensional spectrum divide the stages of human IDD based on levels of amino acid residues and macromolecular compounds, highlighting the specificity of the metabolism process at different stages. The phase difference of metabolites can be explained by molecular biology regulation mechanism. Further studies should explore whether NMR analysis results are consistent with the corresponding MRS quantitative results and whether the macrobiological process can be explained by DTI.

Conventional NMR, DTI, and MRS were used to describe the classification of human IVDs from three levels including anatomical shape, water molecule pathophysiological behavior, and metabolic level adjustment. The process of degeneration was verified through metabolomics analysis.

T_2_WI-weighted images of the IVDs showed that the imaging features of the IVDs of each grade conformed to the Pfirrmann classification. In addition, imaging confirmed that there was no objection to the sorting of human IVD models. This study used functional nuclear magnetism to observe water metabolism based on morphological changes of T_2_WI [54]. Decrease in activity of the high signal of DTI water molecule resulted in changes in the corresponding ADC value and FA value. The ADC value shows that as the degeneration intensifies, the movement of water molecules in the NP is limited and the content decreases, indicating that large amounts of water-fixing groups are lost. Threonine, which contains a large amount of hydroxyl groups, is the key substance for water fixing. The FA value gradually increased with progression in degeneration, implying that the irregular structure and nondirectional distribution tissues that limit dispersion of water molecules increased. These findings indicate an increase in the fibrosis process of the NP tissue in the later stage of degeneration.

Qualitative analysis of NMR showed that the metabolites in the metabolic pool of the IVD were stable and the overall metabolic profile was consistent. Notably, lactate ratio represented accumulation of Lac in the unit structure substance, and its accumulation shows a decrease in energy utilization efficiency in the late stage of degeneration and that the sugar utilization pattern in mass spectrometry metabolomics was changed from the previous glycolysis. In addition, change in metabolites showed transformation from xenobiotics and pentose phosphate to fructose and mannose pathways in the late stages. N-Acetyl resonance compound is an important identification peak of MRS. N-Acetyl/Carb is interpreted as the ratio of collagen content to carbohydrate and hydrocarbons. Carbohydrate content is a marker of matrix structure and carbohydrate metabolism of proteoglycans. The significant decrease in the ratio value indicates decrease of the collagen content in the structural material, which is consistent with the findings on the progressive fibrosis of the NP tissue. On the other hand, N-Acetyl/Cho represents the proportional relationship between the collagen content and the density of the IVD NP cells. The ratio of IDD showed significant decrease indicating that the unit number of cells matching ECM decreased with progress in degeneration. This implies that the proportion of NP cell apoptosis and degeneration increases, and its secretory function decreases. N-Acetyl/Cr represents the proportional relationship between collagen content and energy buffer mechanism, because Cr is an important substance for ATP buffering [55] and it mainly exists as an energy storage form. In the early stage of degeneration, the ratio is high. The downward trend and the abnormal increase in the end stage show the change in energy supply mode at the end of degeneration [56]. Furthermore, the peak of choline compound is used to measure the state of membrane phospholipids, reflecting the density of the NP cells of the IVD. The ratio of Pfirrmann I disc to Pfirrmann II disc in Cho/Carb increased gradually. However, the ratio decreased in Pfirrmann III of the IVD with the lowest value observed in Pfirrmann V disc. This change reflects increase in the ratio of degeneration and apoptosis of NP cells during the degeneration process. In addition, it indicates the decrease in activity of phospholipid synthesis and redox reactions of IVD cells. Therefore, there may be a period of active proliferation of NP cells before degeneration. *In vitro* metabolomics were linked with *in vivo* imaging mimics through spectrogram identification and spectral peak analysis. These findings were then confirmed through histopathological and molecular biology analysis.

## 5. Conclusion

In summary, this study described the relatively complete IDD through correlation analysis of rat and human metabolomics (mass spectrometry metabolomics and NMR metabolomics) combined with imaging, molecular biology, and histopathology verification. The findings of this study show tissue metabolism during degeneration. The findings showed that the process of IDD is closely related to the functional state of the Gly-Ser-Thr metabolic axis. Changes in carbohydrate utilization patterns around this metabolic axis and reduction of the antioxidant capacity of the IVD environment promote degeneration. The process eventually leads to disintegration of the annulus fibrosus and loss of water-fixing groups.

## Figures and Tables

**Table 1:** Differential metabolic pathway table of the three groups of samples.

Comparison group	Metabolic pathway
A group vs. B group	(1) Glutathione metabolism (2) Inositol phosphate metabolism (3) Glycine, serine, and threonine metabolism (4) Purine metabolism (5) Glycolysis/gluconeogenesis (6) Amino acid and nucleotide sugar metabolism (7) Phosphatidylinositol signaling system (8) Cysteine and methionine metabolism (9) Interconversion of pentose and glucuronic acid

A group vs. C group	(1) Glutathione metabolism (2) Cysteine and methionine metabolism (3) D-Glutamine and D-glutamate metabolism (4) Purine metabolism (5) Inositol phosphate metabolism (6) Glycine, serine, and threonine metabolism (7) Aminoacyl tRNA biosynthesis (8) Amino acid and nucleotide sugar metabolism (9) Alanine, aspartic acid, and glutamate metabolism

B group vs. C group	(1) D-Glutamine and D-glutamate metabolism (2) Alanine, aspartic acid, and glutamate metabolism (3) Arginine biosynthesis (4) Arginine and proline metabolism

**Table 2:** General information of human IVD research subjects.

	Pfirrmann I	Pfirrmann II	Pfirrmann III	Pfirrmann IV	Pfirrmann V
GE 3.0 T	28	32	64	121	137
SIEMENS 3.0 T	14	22	52	65	72

## Data Availability

The datasets used and/or analyzed during the current study are available from the corresponding author on reasonable request.
